# Does the timing of postprandial glucose monitoring affect the obstetric and neonatal outcomes in patients with gestational diabetes? A prospective study comparing 1 and 2-h postprandial monitoring

**DOI:** 10.1007/s00404-024-07803-9

**Published:** 2024-11-04

**Authors:** Noa Ben Shoshan, Yossi Mizrachi, Liliya Tamayev, Tal Ben-Ari, Eran Weiner, Giulia Barda

**Affiliations:** 1 Departments of Obstetrics and Gynecology, The Edith Wolfson Medical Center, Holon, Israel; 2 Pediatric Endocrinology Unit, The Edith Wolfson Medical Center, Holon, Israel; 3 Faculty of Medicine, Tel-Aviv University, Tel Aviv, Israel

**Keywords:** Postprandial glucose monitoring, OGTT, GCT, Birth weight, Macrosomia, Shoulder dystocia, Glycemic control, Mode of delivery, Gestational age at delivery, Patient satisfaction, Satisfaction rates, Personal preference

## Abstract

**Objective:**

The aim of this study was to examine the obstetrical and neonatal outcomes in patients with gestational diabetes mellitus (GDM) who had postprandial glucose monitoring 1 vs. 2 h following meals.

**Study design:**

In this prospective cohort study, we included patients with GDM who were referred to our medical center between July 2019 and June 2021. Patients chose the timing of postprandial glucose monitoring based on their own preferences. Obstetrical and neonatal outcomes, as well as patient satisfaction, were compared between patients who performed postprandial glucose monitoring 1 and 2 h after meals (PPG1 vs. PPG2). The primary outcome was birth weight. The study was powered to detect a 250 g increase in birth weight.

**Results:**

Overall, 99 patients were included: 50 in the PPG1 group and 49 in the PPG2 group. Baseline characteristics were comparable between the groups. Neonates in the PPG1 and PPG2 groups had similar birth weights (3319 ± 355 vs. 3319 ± 520 g, respectively, p = 0.99). Glycemic control, mode of delivery, gestational age at delivery, and satisfaction rates were also similar between the study groups.

**Conclusion:**

In patients with GDM, performing1 vs. 2 h following meals resulted in similar obstetrical and neonatal outcomes and similar satisfaction rates. We therefore recommend counseling patients to choose either strategy based on their personal preference.

## What does this study add to the clinical work

**Table:** 

We believe this study will be of interest and practical use to readers as it will permit more flexible and suited follow-up for patients with gestational diabetes mellitus.	

## Introduction

Gestational diabetes mellitus (GDM) is a condition in which carbohydrate intolerance develops or is first diagnosed during pregnancy. Neonates born to women with GDM are at increased risk of macrosomia and other complications [1–6]. A large original randomized trial found that glycemic control was associated with a significant reduction in the rate of serious newborn complications such as birth trauma, shoulder dystocia and perinatal death [7]. The US Preventive Services Task Force meta-analysis of randomized trials also found a reduction in preeclampsia, birth weight > 4000 g and shoulder dystocia in women with GDM treated appropriately compared with no treatment [8]. Therefore, GDM patients receive nutritional counseling and are advised to self-monitor their blood glucose levels. Intensive glucose self-monitoring was associated with lower rates of macrosomia, cesarean deliveries, shoulder dystocia and stillbirth, and a shorter stay in the neonatal intensive care unit, when compared with no intervention [9–11].

Based on the data available, patients are advised to perform glucose self-monitoring four times a day, once after fasting and after each main meal. Assessment of postprandial glucose can be undertaken at either 1 or 2 h after meals. The American Diabetes Association (ADA) and The American College of Obstetricians and Gynecologists (ACOG) recommend aiming for postprandial glucose levels below 140 mg/dl at 1 h (1 h) or 120 mg/dl at 2 h (2 h) to reduce the risk of macrosomia [12]. Although these recommendations are frequently used in common practice, only a few studies have examined the pregnancy and neonatal outcomes in patients performing 1-h compared to 2-h postprandial measurements [13–16]. No study to date has demonstrated a superiority of either approach. Previous studies have focused primarily on glycemic control, while this study adds value by incorporating a large amount of glucose measurements of glucose levels and frequent clinical visits in correlation to clinical outcomes (such as birth weight, mode of delivery and neonatal complications). Furthermore, our study examined patient satisfaction regarding glucose monitoring and the impact on daily life, allowing personalized recommendations based on patient preference. Considering the little evidence available on this important clinical question, we aimed to compare the pregnancy and neonatal outcomes between 1-h (PPG1) and 2-h (PPG2) postprandial glucose monitoring, as well as patient satisfaction.

## Methods

This was a prospective clinical trial conducted between July 2019 and June 2021 at a single tertiary medical center. Women with GDM were eligible for inclusion. In Israel, GDM is diagnosed by a two-step approach; all pregnant women are screened at 24–28 weeks with a 50 g 1-h glucose challenge test (GCT). If the GCT is positive (> 140 mg/dL), patients undergo a 100 g 3-h oral glucose tolerance test (OGTT), based on the Carpenter–Coustan criteria [17]. Patients with pre-gestational diabetes, multiple gestation or chronic diseases were excluded from the study. Patients who were not compliant with regular follow-up visits were also excluded.

At their first visit to the obstetrical care unit, patients were seen by a dietitian and placed on an appropriate diet of 40% carbohydrates, 20% protein and 40% fat. Patients were instructed to self-monitor and document their blood glucose levels using a glucose meter four times a day (fasting and at 1 or 2 h after the onset of each meal according to their own preference). The glucose target levels were based on ACOG recommendations: fasting < 95 mg/dL, PPG1 < 140 mg/dL and PPG2 < 120 mg/dl [15]. For the study, patients were divided into two groups: 1-h postprandial glucose monitoring group (PPG1) and 2-h postprandial glucose monitoring group (PPG2). Recruitment of sequential patients continued until reaching a sufficient preset sample size with a 1:1 ratio.

Recruited patients completed a form containing questions about themselves in regard to their family and physical history, and habits such as smoking and exercise.

Insulin or oral medications were started when more than 30% of the blood glucose values were above the target glucose thresholds. Patients who achieved glycemic control with an appropriate diet only were classified as GDMA1. Patients who needed insulin or oral medications were classified as GDMA2. In our center, patients with GDM are seen every 2 weeks from the time of diagnosis until 32 weeks, and every 1 week afterward until birth. In our department, delivery is planned from week 37.0 until week 39.0 considering the estimated fetal weight and glycemic control.

The primary outcome measure was birth weight.

Secondary outcomes were glycemic control at the last prenatal visit at our clinic, mode of delivery, gestational age at delivery, admission to the neonatal intensive care (NICU) and level of satisfaction.

Large for gestational age (LGA) was defined as birth weight larger than the 90th centile for gestational age according to the local charts. Macrosomia was defined as birth weight greater than 4000 g. Gestational age was confirmed using a first trimester ultrasound scan.

Patients were asked to fill in a satisfaction questionnaire in the last prenatal visit before giving or planning birth, between 36 and 38 weeks of gestation. The questionnaire included the following four questions: Was it difficult to monitor your glucose levels during working hours? Did the timing of glucose monitoring necessitate delaying your usual sleep schedule? To what extent did glucose monitoring disrupt your daily routine? Were any of your activities postponed due to the need to monitor your glucose levels?

Every answer was graded between 0 (no impact) and 3 (significant impact).

Statistical analysis was performed using SPSS software version 28.0 (IBM Corp, Armonk, NY). Continuous variables were presented as mean ± SD and categorical variables were presented as number and percentages. Comparison of continuous variables was performed using the Student’s t test, and comparison of categorical variables was performed using the Chi-square test. Statistical significance was set at *p* < 0.05. The study was powered to detect a 25% difference in the primary outcome. Birth weight was expressed both in grams and percentiles, the latter using the Israeli Society of Obstetrics and Gynecology standard curves—Dollberg curves [18]. A subgroup analysis was performed to compare the two monitoring approaches in GDMA1 and GDMA2 patients separately. On a preliminary check, we found that the mean birth weight in patients with GDM in our center was approximately 3300 ± 400 g. Based on that, we calculated that a sample size of 40 patients in each group would be sufficient to detect a 250 g increase in birth weight, with power of 80% and alpha of 0.05. Ten more patients were recruited in each group to account for loss to follow-up.

The study was approved by the Institutional Ethics Committee (No: 0107-18-WOMC). All patients signed an informed consent before enrollment.

## Results

**Fig. 1:**
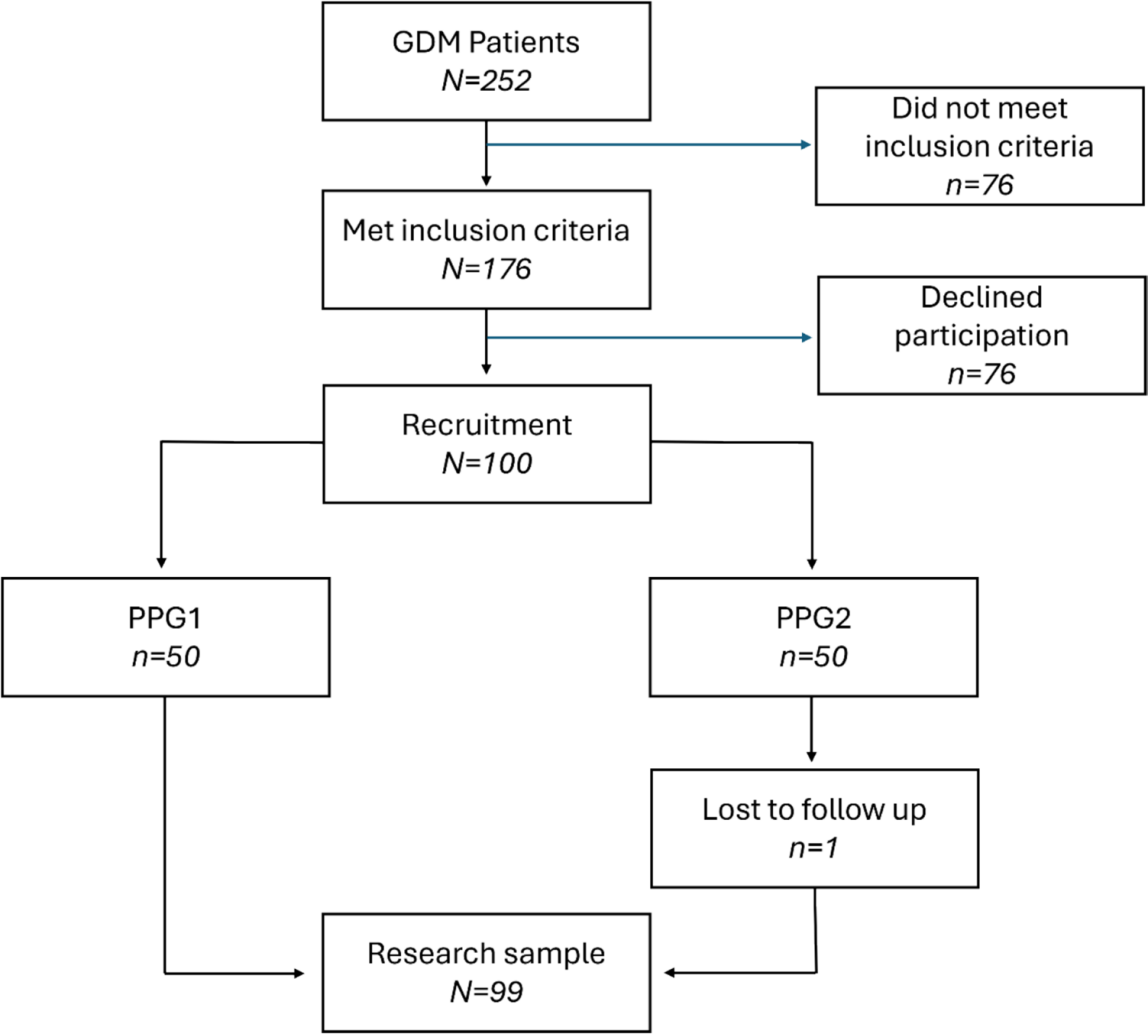
Flowchart of the study recruitment process. GDM, gestational diabetes mellitus; PPG1, 1-h postprandial glucose monitoring; PPG2, 2-h postprandial glucose monitoring

Overall, 100 women were recruited, 50 preferred 1-h postprandial monitoring and 50 preferred 2-h postprandial monitoring. One woman was lost to follow-up (Fig. 1). Finally, data from 99 women were analyzed: 50 women in the PPG1 group and 49 in the PPG2 group. The mean age was 33.0 ± 5.2 years. The nulliparity rate was 35% in the study population. Baseline characteristics were comparable between the study groups (Table 1). Perinatal outcomes according to study group are presented in Table 2. Glycemic control, mode of delivery, gestational age at delivery, birth weight and macrosomia rates were similar between the study groups.

**Table 1:** Baseline characteristics of patients with gestational diabetes according to the timing of postprandial glucose monitoring

	1-h group (*n* = 50)	2-h group (*n* = 49)	*p* value
Age (years)	32.6 ± 5.1	33.4 ± 5.3	0.43
BMI (kg/m^2^)	30.1 ± 5.9	31.9 ± 5.3	0.13
Number of years of education	13.0 ± 2.8	12.7 ± 3.0	0.66
Nulliparity	18 (36.0)	17 (34.7)	0.89
Type of diabetes mellitus			
Gestational diabetes mellitus A1	36 (72.0)	30 61.2)	0.29
Gestational diabetes mellitus A2	14 (28.0)	19 (38.8)	
History of GDM in previous pregnancy	13 (26.0)	28 (28.6)	0.77
Exercise	8 (16.0)	8 (16.3)	0.96
Smoking	3 (6.0)	2 (4.1)	1.00
Family history of diabetes mellitus	20 (40.0)	23 (46.9)	0.48
GCT result (mg/dl)	169 ± 26	163 ± 21	0.35
OGTT 0 (mg/dl)	93 ± 15	92 ± 13	0.75
OGTT 60 (mg/dl)	201 ± 24	191 ± 25	0.08
OGTT 120 (mg/dl)	163 ± 36	164 ± 32	0.96
OGTT 180 (mg/dl)	110 ± 34	118 ± 30	0.26
Hypertensive disorders	2 (4.0)	5 (10.2)	0.26

Data are presented as mean ± standard deviation or n (%)

*GDM* gestational diabetes mellitus, *GCT* glucose challenge test, *OGTT* oral glucose tolerance test

**Table 2:** Perinatal outcomes in patients with gestational diabetes according to the timing of postprandial glucose monitoring

	1-h group (*n* = 50)	2-h group (*n* = 49)	*p* value
Glycemic control (balanced)	41 (82.0)	39 (79.6)	0.76
Induction of labor	17 (34.0)	21 (42.9)	0.36
Delivery mode			
Normal vaginal delivery	22 (44.0)	30 (61.2)	0.14
Cesarean section	26 (52.0)	16 (32.7)	
Vacuum extraction	2 (4.0)	3 (6.1)	
Gestational age at delivery (weeks)^a^	38.2 ± 1.0	37.7 ± 1.5	0.09
Postpartum hemorrhage	0	1 (2.0)	0.49
Preeclampsia	1 (2.0)	3 (6.1)	0.36
Placental abruption	0	0	
Birth weight (g)	3319 ± 355	3319 ± 520	0.99
Birth weight percentile	62.4 ± 24.7	67.0 ± 23.4	0.35
Macrosomia (birth weight > 4000 g)	0	3 (6.1)	0.11
LGA (birth weight ≥ 90th percentile)	9 (18.8)	9 (19.6)	0.92
Cord pH	7.31 ± 0.07	7.32 ± 0.06	0.80
Neonatal complications			
None	32 (64.0)	26 (53.1)	0.27
Hypoglycemia	11 (22.0)	17 (34.7)	
Jaundice	0	1 (2.0)	
NICU admission	2 (4.0)	4 (8.2)	
Other	5 (10.0)	2 (4.1)	

Data are presented as mean ± standard deviation or n (%)

*NICU* neonatal intensive care unit

There were no differences in the rates of induction of labor, post-partum hemorrhage, preeclampsia and placental abruption between the groups.

Women in both groups were comparable with respect to the satisfaction survey (Table 3). About 60% of women in both groups did not have any difficulty monitoring the glucose level during work time and about half of them did not need to change their daily routine or postpone any scheduled activity to be available for glucose monitoring.

**Table 3:** Satisfaction survey in patients with gestational diabetes according to the timing of postprandial glucose monitoring

	1-h group (*n* = 50)	2-h group (*n* = 49)	*p* value
Q1			
None	33 (66.0)	29 (59.2)	0.71
Mild	5 (10.0)	4 (8.2)	
Moderate	6 (12.0)	6 (12.2)	
Severe	6 (12.0)	10 (20.4)	
Q2			
None	38 (76.0)	33 (67.3)	0.22
Mild	5 (10.0)	8 (16.3)	
Moderate	5 (10.0)	2 (4.1)	
Severe	2 (4.0)	6 (12.2)	
Q3			
None	25 (50.0)	26 (53.1)	0.97
Mild	12 (24.0)	10 (20.4)	
Moderate	9 (18.0)	9 (18.4)	
Severe	4 (8.0)	4 (8.2)	
Q4			
Never	29 (58.0)	28 (57.1)	0.36
A few times	8 (16.0)	7 (14.3)	
Sometimes	12 (24.0)	9 (18.4)	
Many times	1 (2.0)	5 (10.2)	

Q1 Was it difficult to monitor your glucose levels during working hours?

Q2 Did the timing of glucose monitoring necessitate delaying your usual sleep schedule?

Q3 To what extent did glucose monitoring disrupt your daily routine?

Q4 Were any of your activities postponed due to the need to monitor your glucose levels?

On subgroup analysis, birth weight was still similar between the PPG1 and PPG2 groups when analyzing only GDMA1 patients (3309 ± 375 vs. 3322 ± 617 g, respectively, *p* = 0.91) and only GDMA2 patients (3308 ± 328 vs. 3231 ± 481 g, respectively, *p* = 0.60) separately.

## Discussion

Principal findings: In the current study, we compared the pregnancy and neonatal outcomes in GDM patients who had postprandial glucose monitoring either 1 or 2 h following meals. We found all outcomes to be comparable between the study groups. Glycemic control and birth weight were comparable, as well as patient satisfaction.

Results in the context of what is known: To compare the rate of abnormal glucose levels between PPG1 and PPG2, Sivan et al. in 2001 enrolled 68 women with diet-controlled GDM, who had their blood glucose levels measured 1 h and 2 h postprandially for 1 week. Unlike our results, in this study, the rate of abnormal PPG1 was 2.5-fold greater than PPG2 after breakfast, in contrast to dinner, where PPG2 was twofold higher. The explanation for the discrepancy between morning and evening measurements might be explained by differences in meal composition and physical activity. The authors did not report perinatal outcome; therefore, it is not possible to determine whether the difference between morning and evening had any impact on perinatal outcome [15]. Moses RG was the first in 1999 to compare selected pregnancy outcomes in women with GDM whose glucose level was tested by 1 h or 2 h postprandially. In agreement with our results, birth weight, percentage of women requiring insulin, and the total daily dose of insulin were similar in both groups. Women monitored by 1 h postprandially had fewer large for gestational age (LGA) infants and a reduced rate of emergency cesarean sections, suggesting the possibility that PPG1 monitoring and treatment adjustment accordingly may result in a better outcome. However, the total numbers were small and the data should be confirmed in a larger cohort [14]. Another study compared adverse perinatal outcome in 112 women with GDM monitored 1 vs. 2 h postprandially. In line with our results, the authors found that gestational age at delivery, birth weight and percentile were similar in both groups. Unlike our results, Insulin therapy was initiated more frequently in the 2-h group, but it did not significantly influence the rates of macrosomia, LGA and cesarean Sects.  [13]. In our study, the rate of macrosomia was very small (no cases in the PPG1 group and only 3 cases, 6.1%, in the PPG2 group). This data might reflect our weekly follow-up from 32 weeks and active management of inducing labor when macrosomia is suspected by ultrasound fetal weight estimation. A recent study examined the correlation between PPG1 and PPG2 and adverse perinatal outcomes. Although in that study, PPG1 and PPG2 were measured only during the morning of each perinatal visit with the patient serving as their own control, the results, similar to ours, indicated that neither PPG1 nor PPG2 measurements were predictive of perinatal complications [16].

Clinical Implications: Furthermore, in the current study, patients were asked to fill in a satisfaction questionnaire which included four questions aimed at providing information about difficulties in monitoring. There was no difference in satisfaction survey between the PPG1 and PPG2 monitoring groups. Most (60%) women in both groups reported no difficulty in monitoring their blood glucose levels. Furthermore, in each group, more than half of the participants did not claim any need to change their scheduled or routine activity. This provides evidence of good compliance with either approach (PPG1 and PPG2) and encourages physicians to counsel GDM patients on both options and let them choose according to their own preference.

Strengths and limitations: Our study has some strengths. First, it is a single-center study in which all patients were treated according to standard protocol and followed by the same team in our Maternal and Fetal Medicine Unit. Second, our patients had a long follow-up and frequent perinatal visits, as it started with the diagnosis of GDM and continued every 1–2 weeks until delivery; therefore, our results are based on a large sample of measurements and better reflect the impact of PPG1 and PPG2 on obstetrical and neonatal outcomes, compared with previously published data. Third, this is the first study which provides information about satisfaction of monitoring by PPG1 and PPG2. The limitations of the study are its non-randomized design and relatively small sample size. Furthermore, the study design, in which patients were allocated into one of the study groups in an alternating way, is expected to generate groups with similar baseline characteristics, as evident by Table 1, and this could account for possible confounders.

In conclusion, our findings suggest that in patients with gestational diabetes, postprandial glucose monitoring after 1 or 2 h results in similar neonatal outcomes and patient satisfaction. Considering these results, we recommend counseling women and let them choose either strategy according to their personal preference.

## Data Availability

The data that support the findings of this study are available from the corresponding author, upon reasonable request.
